# Risk factors for implant-related fractures after proximal femoral osteotomy in children with developmental dysplasia of the hip: a case-control study

**DOI:** 10.1080/17453674.2020.1848315

**Published:** 2020-11-24

**Authors:** Jing Ding, Zhen-Zhen Dai, Zhu Liu, Zhen-Kai Wu, Zi-Ming Zhang, Hai Li

**Affiliations:** Department of Pediatric Orthopedics, Xin Hua Hospital affiliated to Shanghai Jiao Tong University School of Medicine, Shanghai, China

## Abstract

Background and purpose — Proximal femoral osteotomy (PFO) is commonly performed to treat children with developmental dysplasia of the hip (DDH). Implant-related femoral fractures after osteotomy are sometimes reported, but the potential risk factors for these fractures remain unclear. We investigated the association of implant-related fractures with PFO and potential risk factors for these fractures.

Patients and methods — We retrospectively reviewed 1,385 children undergoing PFO for DDH in our institution from 2009 to 2016 after obtaining institutional review board (IRB) approval and identified 27 children (28 hips, fracture group) with implant-related femoral fractures after PFO. We selected 137 children (218 hips, control group) without fractures who matched the children in the fracture group by age, weight, surgeon, and surgical period. Relevant clinical data were collected and compared between the 2 groups. Multiple analyses of risk factors for implant-related fractures were conducted by logistic regression with the stepwise regression method.

Results — The occurrence rate of implant-related fractures was 1.9% (27/1,385). Compared with the control group, the fracture group more commonly exhibited bilateral involvement (74% vs. 53%, p = 0.04), used a spica orthosis for immobilization after osteotomy (43% vs 21%, p = 0.01) and exhibited mild remodeling at the osteotomy site (46% vs. 19%, p = 0.003), and less commonly required capsulotomy during osteotomy (61% vs. 79%, p = 0.03). According to the multiple regression analysis, the only factor identified as an independent risk factor for implant-related fractures was mild remodeling at the osteotomy site (OR = 3.2, 95% CI 1.4–7.5). Remodeling at the osteotomy site was significantly associated with varus osteotomy (coefficient = 1.4, CI 1.03–1.8). The fracture occurred at a mean of 12 months (2.2–25) after osteotomy or 3.3 months (0–12) after implant removal. In children undergoing implant removal, the fractures mostly occurred at the osteotomy site (n = 13/15), while in those with the implant remaining, the fractures mostly occurred in the screw hole (n = 8/13).

Interpretation — The type of PFO performed is not associated with implant-related fractures in children with DDH. Children with mild remodeling at the osteotomy site should be closely followed up, regardless of whether the hardware is removed, and high-intensity activity should not be permitted until moderate or extensive remodeling is confirmed. After PFO, the implants should be removed when solid union is achieved at the osteotomy site.

Proximal femoral osteotomy (PFO) is commonly performed to correct proximal femoral deformities in individuals with developmental dysplasia of the hip (DDH), and types of PFO include femoral shortening, varus osteotomy, and derotation osteotomy. Internal fixation implants, such as a blade plate or locking compress plate, are used to maintain the stability of the osteotomy site (Papavasiliou and Papavasiliou 2005, Sharpe et al. 2006, Shaw et al. 2016). Implant-related complications or fractures after osteotomy have been reported, with a prevalence rate of 0.3% to 3.6% (Jain et al. 2012, 2016). Although the rate is low, these complications or fractures prolong immobilization in children and sometimes require additional surgery. Few studies have investigated relations between implant-related fractures and sites of fractures or types of plates (Becker et al. 2012, Jain et al. 2012, 2016, Chung et al. 2018). Jain et al. (2012) reported that the femur is more likely to incur an implant-related fracture than are other bones, and suggested that the level of stress exerted by an implant can be high over short anatomic distances in the proximal femur. However, the authors did not stratify the results by the indications for PFO, such as DDH, Perthes disease, and cerebral palsy. Varus in PFO may increase the level of stress on the implant, which in turn leads to stress shielding at the osteotomy site; therefore, it is presumable that changes in both the anatomy of the proximal femur and stress on the implant and osteotomy site may increase the probability of implant-related fractures. However, the relation of PFO itself to implant-related fractures after DDH has not been clarified, and the potential risk factors for implant-related fractures remain unclear. Therefore, we investigated the association of implant-related fractures with PFO and possible risk factors for these fractures.

## Patients and methods

**Figure 1.**
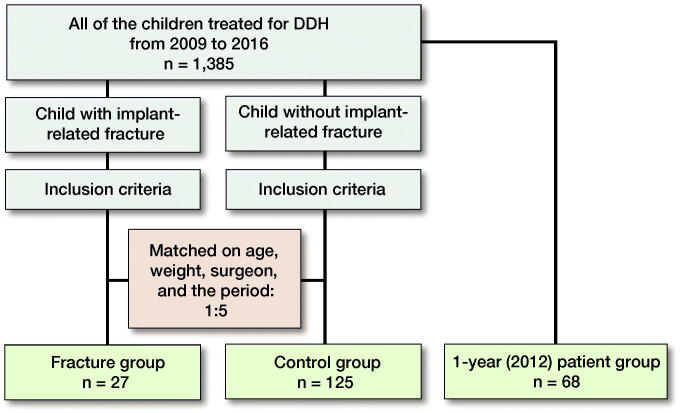
Flow chart of patients reviewed and selected.

We retrospectively reviewed all 1,385 children who were younger than 14 years old, did not have pathological or metabolic diseases or cerebral palsy, and had undergone PFO at our hospital from 2009 to 2016. From this initial patient population, we identified every child who had sustained an implant-related fracture. For each of these children, we selected 5 children who did not have a fracture but were matched in terms of the surgeon who operated on him or her, age (difference < 6 months), weight (difference < 2 kg), and duration since osteotomy (within 1 month) to form a control group. Furthermore, to determine whether the selection of the control group was biased, we collected the demographic data of the children included in the initial patient population (2009–2016) over the period of 1 whole year (January 2012 to December 2012) and compared the data with those of the control group (Figure 1).

The inclusion criteria of the fracture group were as follows: (a) a femoral fracture adjacent to the osteotomy or implant site; (b) a fracture that occurred within 2 years after PFO, regardless of whether the implant was removed; (c) the absence of a history of severe trauma, such as a fall from a height or car accident; and (d) complete medical data spanning a follow-up period of more than 2 years after PFO.

The inclusion criteria of the control group were (a) the absence of a femoral fracture within 2 years after PFO and (b) complete medical data spanning a follow-up period of more than 2 years after PFO.

In our hospital, PFO is an elective procedure performed in combination with pelvic osteotomy in children older than 18 months old with DDH by the senior surgeon according to the method described by Weinstein and Flynn (2014). During PFO, we usually perform femoral derotation and/or varus or shortening osteotomy. Basically, we decrease the anteversion angle to no less than 30°, decrease the neck–shaft angle to no less than 120°, and shorten the femur for complete dislocation. Moreover, capsulotomy is usually performed in children with Tönnis grade III/IV and sometimes those with Tönnis grade II to enable reduction and capsulorrhaphy. The extent of dislocation of the femoral head, neck–shaft angle, and anteversion angle in the proximal femur are evaluated by preoperative radiography and CT.

**Figure 2.**
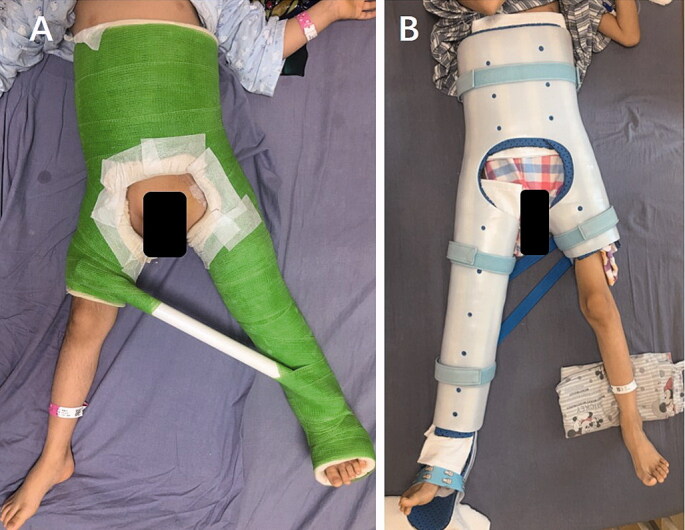
Immobilization types: A = spica cast. B = spica orthosis.

Postoperative immobilization by a spica cast or spica orthosis (Figure 2) is continued for 6–8 weeks, and active non-weight-bearing exercises throughout a range of motion are performed for approximately 3–4 weeks. Thereafter, gradual weight-bearing is permitted when union of the osteotomy site is confirmed by radiography. The implant is removed routinely at approximately 6–12 months after PFO when solid union has occurred. For children with bilateral DDH, we usually perform osteotomy on each side with an interval of 4–6 months between surgeries.

We collected the following demographic and clinical data: age, sex, weight, side (unilateral or bilateral), severity of DDH (dysplasia or dislocation), degree of derotation or varus, length of shortening in PFO, types of implant, whether capsulotomy existed, implant removal status, remodeling condition at the osteotomy site, time to implant removal, follow-up time, fracture site (osteotomy site, screw hole or others), and time from osteotomy to fracture or implant removal.

**Figure 3.**
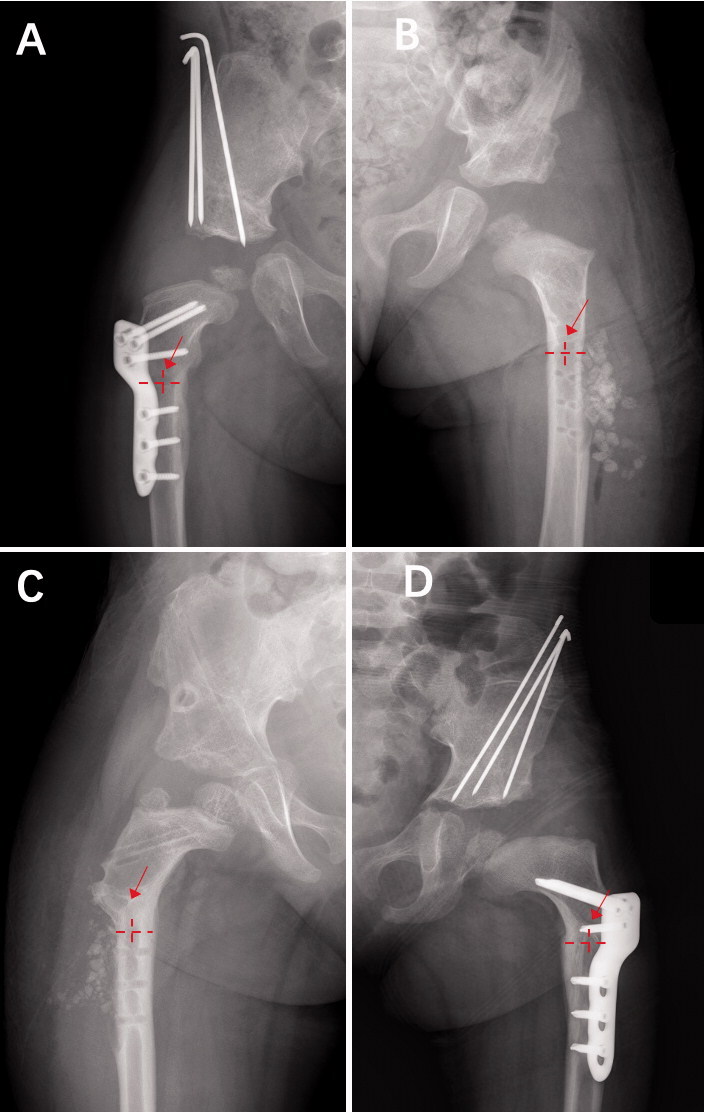
Evaluation of the remodeling condition at the osteotomy site. A, B: Proportion of residual trace line (red arrow) located at the osteotomy site to diameter of the medullary cavity (red dotted line, vertical line represents the center point of the diameter of medullary cavity) was less than Ѕ, so the remodeling condition of the osteotomy site was defined as moderate or extensive. C, D: Proportion of residual trace line (red arrow) located at the osteotomy site to diameter of the medullary cavity (red dotted line, vertical line represents the center point of the diameter of medullary cavity) was more than Ѕ, so the remodeling condition of the osteotomy site was defined as mild.

The time at which the fracture occurred in the fracture group was set as the endpoint of the follow-up period for the children in the control group. Whether the implant was still in situ was also determined at the endpoint. The remodeling condition at the osteotomy site was also evaluated at the endpoint. According to the methods described by Davids et al. (2013), for the convenience of statistical analysis, we classified remodeling radiographically as the proportion of the residual trace line located at the osteotomy site to the diameter of the medullary cavity (Figure 3). If the proportion was smaller than 1/2, a moderate or extensive remodeling condition existed at the osteotomy site; if the proportion was larger than 1/2, mild remodeling existed.

### Statistics

The categorical variables were assessed by chi-square test and Fisher’s exact test, and the continuous variables were computed by t-tests. Possible relations between all kinds of factors, such as demographics (age, sex, weight), diseases (severity of DDH, side), surgical factors (degree of derotation or varus, length of shortening in PFO, types of implant, whether a capsulotomy existed) and postoperative factors (immobilization, remodeling condition at the osteotomy site, implant removal status), were assessed by Spearman rank correlation test and logistic regression. Multiple analyses of risk factors for implant-related fractures were evaluated by logistic regression, and odds ratios (ORs) with their 95% confidence intervals (CIs) were also obtained. The potential risk factors included in the multiple analyses were the type of PFO, factors considered in previous studies (types of implant, implant removal status) (Jain et al. 2012, Chung et al. 2018) and the positive factors identified in the crude analysis that were assumed to have a cause–effect relation. The stepwise regression method was used to find the most appropriate logistical model. Statistical analyses were carried out with the statistical software Stata/SE for Windows (version 15.0; StataCorp LLC, College Station, TX, USA), and all statistical tests were 2-tailed; p-values < 0.05 were considered significant.

### Ethics, funding, and potential conflicts of interest

The study was conducted according to the ethical principles stated in the Declaration of Helsinki. The study received approval from the Institutional Review Board/Ethics Committee of Xin Hua Hospital (reference number: XHEC-D-2019-011). This work was supported by the Shanghai Collaborative Innovation Center for Translational Medicine (grant TM201712) and the Clinical Research Unit, Xin Hua Hospital Affiliated to Shanghai Jiao Tong University School of Medicine (grant 16CR3100B). No conflicts of interest were declared by the authors.

## Results

We identified 27 children (28 hips) with fractures (mean age 5.2 years [2–14]), accounting for a fracture rate of 1.9% (27/1,385). The 125 children (190 hips) in the control group had a mean age of 4.5 years (2–13). The 2 groups were similar in terms of age, sex, and weight (Table 1). The children in the 1-year group selected from the initial study population and children in the control group were similar in terms of sex, age, weight, side, and dislocation (Table 2).

**Table 1.** Patient characteristics stratified by occurrence of implant-related fractures. Values are count (%) or mean (range)

	All subjects	Fracture	Non-fracture	
Characteristics	(N = 152)	(n = 27)	(n = 125)	p-value
Female sex	129 (82)	22 (81)	107 (82)	0.6
Age, year	4.6 (2–14)	5.2 (2–14)	4.5 (2–13)	0.3
Weight, kg	19.6 (8–66)	21.4 (9.5–66)	19.2 (9.4–54)	0.4
				
				
				

**Table 2.** Comparison of patient characteristics between control group and 1-year patient group. Values are count (%) or mean (range)

	Control	1-year patient	
	group	group	
Characteristics	(n = 125)	(n = 68)	p-value
Female sex	107 (82)	53 (78)	0.2
Age, year	4.5 (2–13)	4.6 (2–14)	0.8
Weight, kg	19.2 (9.4–54)	18.0 (8–50)	0.3
Side			0.1
Unilateral	59 (47)	24 (35)	
Bilateral	66 (53)	44 (65)	
Dislocation	86 (69)	52 (76)	0.3

Compared with the control group, the fracture group more commonly had bilateral involvement (74% vs. 53%, p = 0.04, Table 1), used a spica orthosis for immobilization after osteotomy (43% vs. 21%, p = 0.01), and exhibited mild remodeling at the osteotomy site (46% vs. 19%, p = 0.003) but less commonly required capsulotomy during osteotomy (61% vs. 79%, p = 0.03). However, no significant differences between the two groups were found in other clinical factors, such as dislocation, derotation, varus, shortening osteotomy in the proximal femur, implant type, implant side, or time to implant removal (Table 3)

**Table 3.** Clinical characteristics of the hip stratified by occurrence of implant-related fractures. Values are count (%) or mean (range)

	All subjects	Fracture	Non-fracture	
Characteristics	(N = 218)	(n = 28)	(n = 190)	p-value
Severity of DDH				1.0
Dysplasia	69 (32)	9	60 (32)	
Dislocation	149 (68)	19	130 (68)	
Derotation ^a^	3.3 (0–8)	3.6 (0–6)	3.2 (0–8)	0.2
Varus ^a^	4.5 (0–8)	4.8 (1–7)	4.4 (0–8)	0.2
Shortening, cm	1.3 (0–5)	1.3 (0–4)	1.3 (0–5)	1.0
Hardware				0.8
Locking plate	167 (77)	21	146 (77)	
Blade plate	51 (23)	7	44 (23)	
Immobilization				0.01
Spica cast	166 (76)	16	150 (79)	
Spica orthosis	52 (24)	12	40 (21)	
Capsulotomy	168 (77)	17	151 (79)	0.03
Implant removed				0.4
No	117 (54)	13	104 (55)	
Yes	101 (46)	15	86 (45)	
Remodeling condition at the osteotomy site		0.003		
Moderate or				
extensive	169 (78)	15	154 (81)	
Mild	49 (22)	13	36 (19)	
Months to implant				
removal ^b^	10 (5–21)	11 (7–21)	10 (5–20)	0.4

**^a^**1 means 5°.

**^b^** In children who had the implants removed.

**Figure 4.**
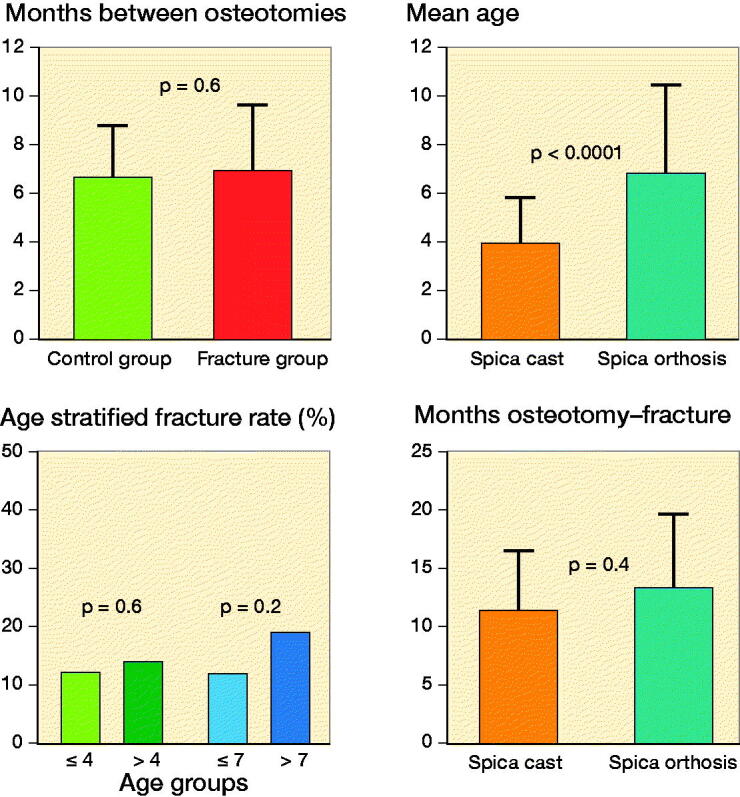
A: Comparison of interval from separate osteotomy surgery in the children with bilateral DDH between fracture and control groups (6.6 vs. 7 months, p = 0.6). B: Comparison of age of children by immobilization type (4 vs. 6.4 years old, p < 0.0001). C: Fracture rate according to age group (12% vs. 14%, p = 0.6; 11.8% vs. 19%, p = 0.2). D: Comparison of time from osteotomy to fracture between children with different immobilization types (11.4 [SD 5.2] vs. 13.2 [SD 6.4] months, p = 0.4).

For the children with bilateral involvement, the time to the separate osteotomy surgery did not statistically significantly differ between the children with a fracture and those without a fracture (Figure 4A). We also found that age was significantly related to the immobilization type selected (coefficient = 1.4, Table 4), and the children using a spica orthosis were younger than those using a spica cast (average age, 4 vs. 7 years old, Figure 4B). The rate of implant-related fractures was similar between the 4- and 7-year-old groups (Figure 4C). There were no differences in the time from osteotomy to fracture between the children immobilized by an orthosis and those immobilized by a cast (Figure 4D). Capsulotomy was significantly related to dislocation (coefficient = 2.4), age (coefficient = 0.6), and shortening osteotomy (coefficient = 6.4, Table 5). Varus osteotomy was found to be an independent factor for the remodeling condition at the osteotomy site (coefficient = 1.4, Table 6).

**Table 4.** Multiple analyses of factors related to immobilization type by logistic regression

Related factors	Coefficient (95% CI)
Age	1.4 (1.1–1.7)
Side	0.7 (0.4–1.6)
Weight	1.0 (1.0–1.1)
Dislocation	1.6 (0.7–3.6)

CI = confidence interval.

We finally incorporated the following factors into the multifactorial analysis of implant-related fractures (Table 4): age, side, severity of DDH (dysplasia or dislocation), degree of derotation or varus, length of shortening in PFO, types of implant, implant removal status, and remodeling condition at the osteotomy site. According to the stepwise regression results, the factor identified as an independent risk factor for implant-related fractures was mild remodeling at the osteotomy site (OR = 3.2, Table 7).

For the children in the fracture group, the average time from osteotomy to fracture was 12 months (2.2–25 months). It was 9.6 months (2.2–24 months) for the children with implants remaining and 15 months (8.7–25) for the children who underwent implant removal. The average time from implant removal to fracture was 3.3 months (0–12 months) (Table 8).

**Figure 5.**
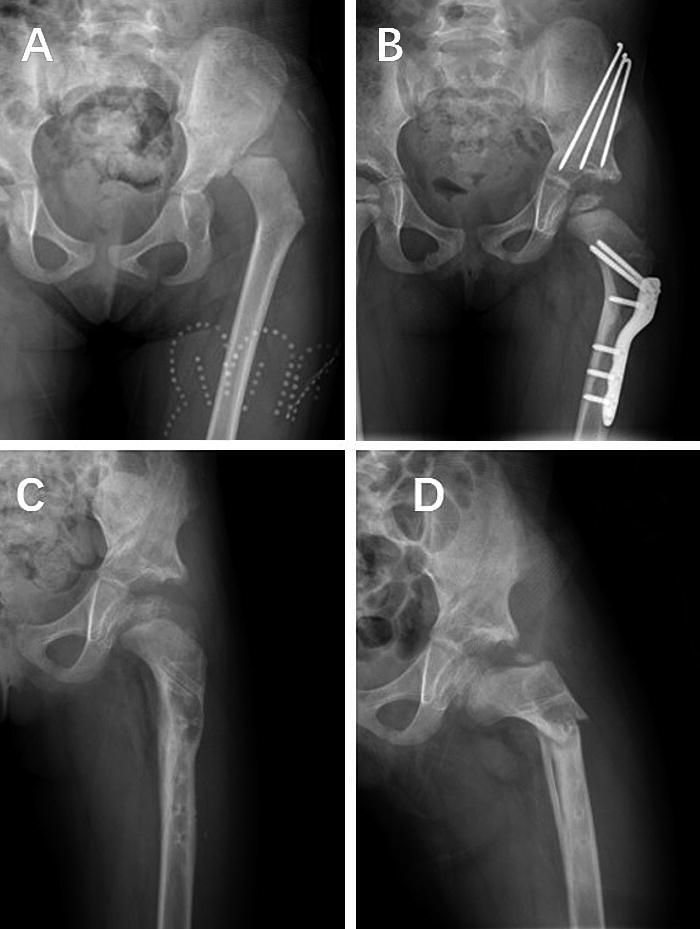
A: A 4-year-old girl with DDH. She underwent PFO, open reduction, and pelvic osteotomy. B: Image taken at 7.5 months postoperatively. C: Implant was removed at 13 months postoperatively. D: She had an implant-related fracture at the osteotomy site at 3.5 months after implant removal when she was walking.

**Figure 6.**
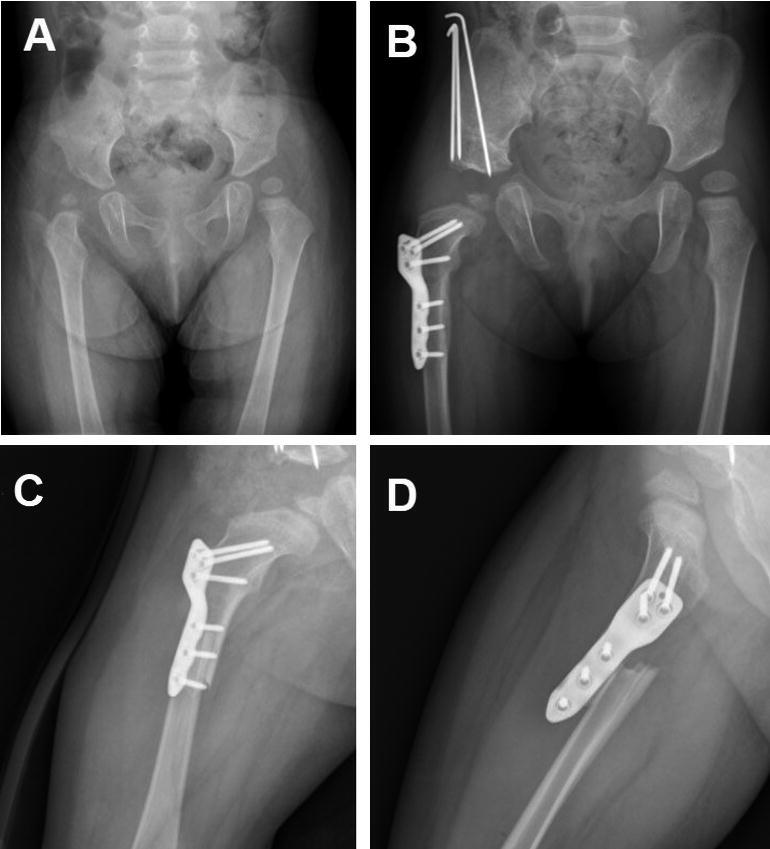
A: A 2-year-old girl with right DDH. She underwent PFO, open reduction, and pelvic osteotomy. B: Image taken at 5 months postoperatively. C, D: She had an implant-related fracture in the screw hole at 10.4 months postoperatively when she was walking and was immobilized with an orthotic.

A fracture occurred at the osteotomy site in 16/28 children. The distribution of fracture sites significantly differed between the children who did and did not undergo hardware removal (p = 0.001, Table 8). In the children who underwent implant removal, the fractures mostly occurred at the osteotomy site (13/15) (case shown in Figure 5), while in those who still had the implant the fractures mostly occurred in the screw hole (8/13, Table 8) (case shown in Figure 6).

## Discussion

We did not find any relations between the type of PFO and implant-related fractures in children with DDH. Only mild remodeling at the osteotomy site was identified as an independent risk factor for these fractures. However, varus osteotomy was found to be related to the remodeling condition. The distribution of fracture sites differed between the children who did and did not undergo hardware removal.

No clear definitions of implant-related fractures exist; generally, these fractures include peri-implant fractures that occur within 6 months after hardware removal without trauma (Busam et al. 2006, Chung et al. 2018). The occurrence rate of implant-related fractures has been reported to be 0.3–3.6% by other authors (Jain et al. 2016, Chung et al. 2018) and was 1.9% (27/1,385) in our study. The rate varied by location and disease. Jain et al. (2012) reported that the femur is more likely to incur an implant-related fracture than are other bones and suggested that the level of stress shielding exerted by the implant can be high in the proximal femur. However, we did not find varus, derotation, or shortening osteotomy in PFO to be associated with implant-related fractures. Although locking plates are thought to reduce the level of stress at the osteotomy site (Bottlang et al. 2010, Becker et al. 2012), neither the occurrence of an implant-related fracture nor the fracture site was found to be related to plate type in our study. Children with DDH always have a larger neck–shaft angle or anteversion angle in the proximal femur, especially those with complete dislocation. Therefore, in children with DDH, PFO is performed to restore the anatomy of the proximal femur to a relatively normal state and, in theory, should not increase the stress in the proximal femur or the implant.

**Figure 7.**
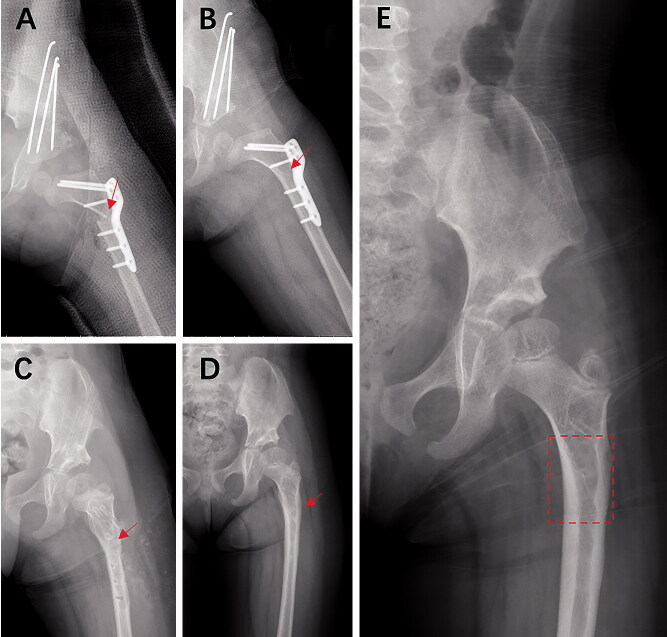
Remodeling process at the osteotomy site in a 4-year-old girl with PFO. A: End-to-end alignment at the osteotomy site immediately after operation (red arrow). B: mild remodeling at the osteotomy site (union already) with a distinct residual line (red arrow) from medial cortex of proximal segment in the medullar cavity (6 months after PFO). C: mild remodeling with obscure residual line (red arrow) (15 months after PFO with hardware removal). D: moderate remodeling at the osteotomy site with obscure and shorter residual line (red arrow) in the medullar cavity (27 months after PFO). E: extensive remodeling with obscure and short residual line in the medullar cavity (red dotted box) (33 months after PFO).

Our study showed that mild remodeling at the osteotomy site may be a risk factor for implant-related fractures. PFO is a kind of “end-to-end” technique with a transverse osteotomy at the subtrochanteric level, after which the proximal segment is abducted to some degree (varus osteotomy) and aligned with the distal shaft segment (Davids et al. 2013). After the union of the osteotomy site, the medial cortex of the proximal segment becomes the residual line in the medullary cavity, as seen in anterior–posterior radiographs, which gradually disappears through remodeling within 2–3 years, according to our observations (Figure 7). The process of bone remodeling follows Wolff’s Law but may be affected by the alignment of the osteotomy site. We found severe varus osteotomy to be related to the remodeling condition at the osteotomy site (Table 6). We think that remodeling at the osteotomy site may be affected not only by the alignment of the osteotomy site during surgery but also by subsequent bone healing and remodeling. Therefore, we do not think that the condition of remodeling at the osteotomy site can be used to measure the extent of varus osteotomy performed. However, how the mechanical characteristics of the proximal femur change with remodeling conditions remains unclear. Whether mild remodeling at the osteotomy site weakens the mechanical strength of the proximal femur, making it susceptible to fracture, needs to be researched further. We also suggest that children with mild remodeling at the osteotomy site, regardless of whether the hardware is removed, should be followed up closely and that high-intensity activity should not be permitted until moderate or extensive remodeling at the osteotomy site is confirmed.

In our institution, implant removal after PFO in children with DDH is performed routinely because Chinese parents usually prefer not to leave any metal implants in their child’s body. In addition, implant removal facilitates future surgeries, if needed (Jain et al. 2012, 2016). In our study, whether the hardware was retained or removed was not identified as a risk factor for implant-related fractures. However, it was found to be related to the location of the fracture site (Table 8), which can be explained as “stress shielding,” as suggested by previous research (Lovell et al. 1999, Hanson et al. 2008, Jain et al. 2012). Therefore, the implants should be removed after PFO when solid union is observed at the osteotomy site.

Our study has several limitations. First, because of its retrospective nature, there may have been discrepancies in the characteristics of the patients between groups (Chung et al. 2018). The case-control design could have decreased the effects of confounders, including age, sex, surgeon, and surgical period. Moreover, the children in the control group did not differ in age, weight, side, or dislocation from those in the one-year group, indicating that the control group is a representative sample of the initial study population. Second, we cannot determine whether clinical factors other than those we recorded, such as the postoperative range of motion in the hip, are related to fractures after osteotomy.

In conclusion, PFO is not associated with implant-related fractures in children with DDH. We suggest that children with mild remodeling at the osteotomy site are followed up closely, regardless of whether the hardware is removed, and that high-intensity activity is not permitted until moderate or extensive remodeling at the osteotomy site is confirmed. After PFO, the implants should be removed when solid union at the osteotomy site occurs.

## Supplementary data

Tables 5–8 are available as supplementary data in the online version of this article, http://dx.doi.org/10.1080/17453674.2020.1848315
